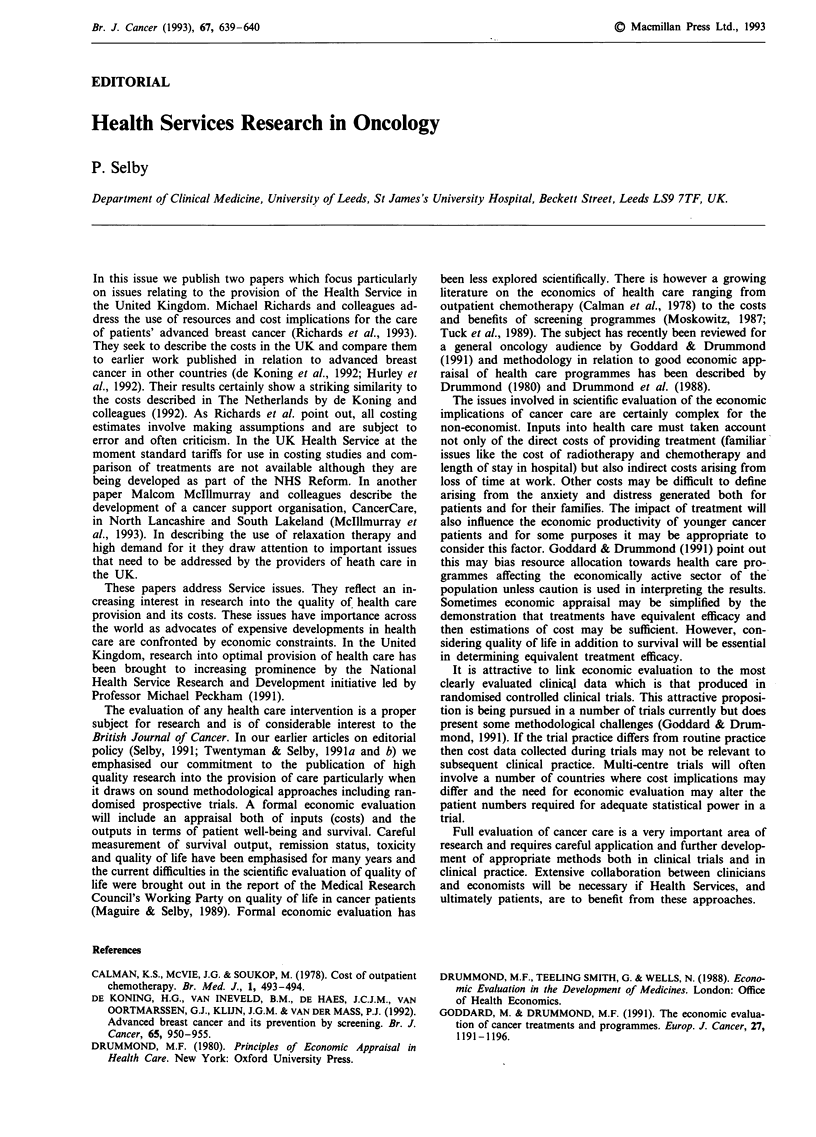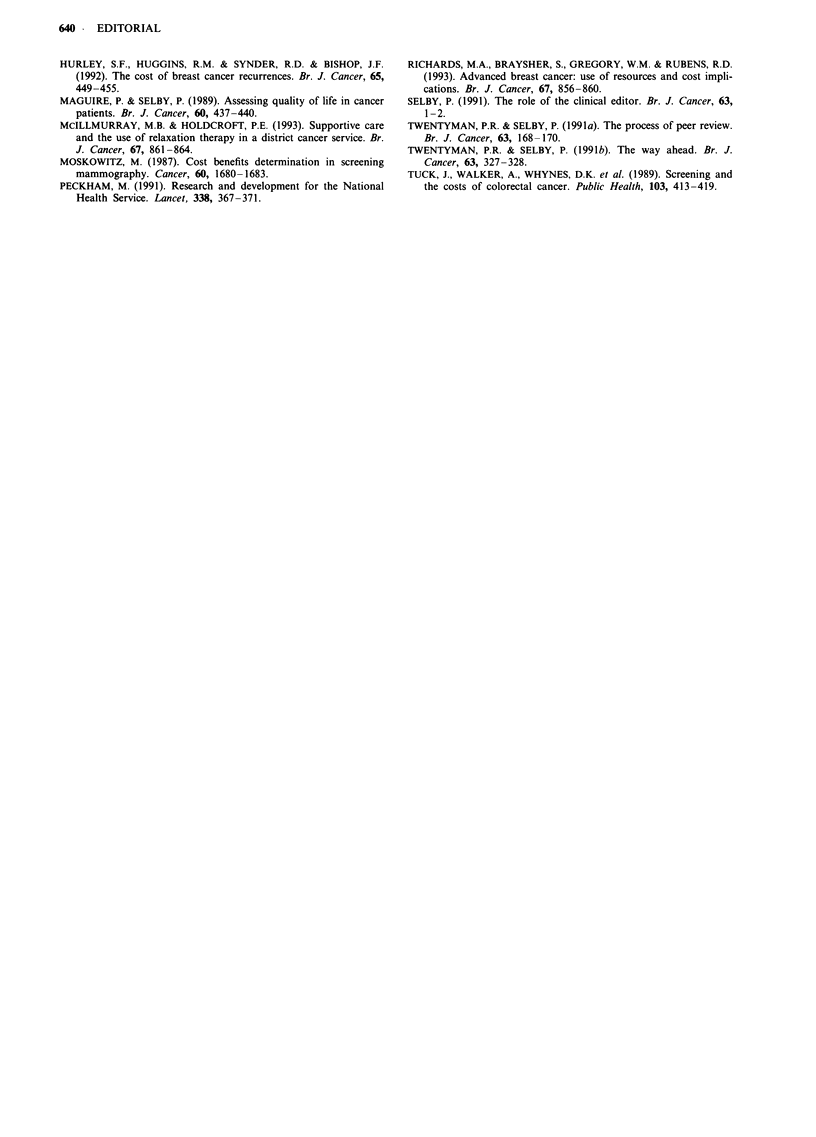# Health services research in oncology.

**DOI:** 10.1038/bjc.1993.118

**Published:** 1993-04

**Authors:** P. Selby


Br. J. Cancer (1993), 67, 639-640 © Macmillan Press Ltd., 1993

**EDITORIAL**

### Health Services Research in Oncology

P. Selby

*Department of Clinical Medicine, University of Leeds, St James's University Hospital, Beckett Street, Leeds LS9 7TF, UK.*

In this issue we publish two papers which focus particularly on issues relating to the provision of the Health Service in the United Kingdom. Michael Richards and colleagues address the use of resources and cost implications for the care of patients' advanced breast cancer (Richards *et al.*, 1993). They seek to describe the costs in the UK and compare them to earlier work published in relation to advanced breast cancer in other countries (de Koning *et al.*, 1992; Hurley *et al.*, 1992). Their results certainly show a striking similarity to the costs described in The Netherlands by de Koning and colleagues (1992). As Richards *et al.* point out, all costing estimates involve making assumptions and are subject to error and often criticism. In the UK Health Service at the moment standard tariffs for use in costing studies and comparison of treatments are not available although they are being developed as part of the NHS Reform. In another paper Malcom McIllmurray and colleagues describe the development of a cancer support organisation, CancerCare, in North Lancashire and South Lakeland (McIllmurray *et al.*, 1993). In describing the use of relaxation therapy and high demand for it they draw attention to important issues that need to be addressed by the providers of heath care in the UK.

These papers address Service issues. They reflect an increasing interest in research into the quality of health care provision and its costs. These issues have importance across the world as advocates of expensive developments in health care are confronted by economic constraints. In the United Kingdom, research into optimal provision of health care has been brought to increasing prominence by the National Health Service Research and Development initiative led by Professor Michael Peckham (1991).

The evaluation of any health care intervention is a proper subject for research and is of considerable interest to the *British Journal of Cancer*. In our earlier articles on editorial policy (Selby, 1991; Twentyman & Selby, 1991*a* and *b*) we emphasised our commitment to the publication of high quality research into the provision of care particularly when it draws on sound methodological approaches including randomised prospective trials. A formal economic evaluation will include an appraisal both of inputs (costs) and the outputs in terms of patient well-being and survival. Careful measurement of survival output, remission status, toxicity and quality of life have been emphasised for many years and the current difficulties in the scientific evaluation of quality of life were brought out in the report of the Medical Research Council's Working Party on quality of life in cancer patients (Maguire & Selby, 1989). Formal economic evaluation has been less explored scientifically. There is however a growing literature on the economics of health care ranging from outpatient chemotherapy (Calman *et al.*, 1978) to the costs and benefits of screening programmes (Moskowitz, 1987; Tuck *et al.*, 1989). The subject has recently been reviewed for a general oncology audience by Goddard & Drummond (1991) and methodology in relation to good economic appraisal of health care programmes has been described by Drummond (1980) and Drummond *et al.* (1988).

The issues involved in scientific evaluation of the economic implications of cancer care are certainly complex for the non-economist. Inputs into health care must taken account not only of the direct costs of providing treatment (familiar issues like the cost of radiotherapy and chemotherapy and length of stay in hospital) but also indirect costs arising from loss of time at work. Other costs may be difficult to define arising from the anxiety and distress generated both for patients and for their families. The impact of treatment will also influence the economic productivity of younger cancer patients and for some purposes it may be appropriate to consider this factor. Goddard & Drummond (1991) point out this may bias resource allocation towards health care programmes affecting the economically active sector of the population unless caution is used in interpreting the results. Sometimes economic appraisal may be simplified by the demonstration that treatments have equivalent efficacy and then estimations of cost may be sufficient. However, considering quality of life in addition to survival will be essential in determining equivalent treatment efficacy.

It is attractive to link economic evaluation to the most clearly evaluated clinical data which is that produced in randomised controlled clinical trials. This attractive proposition is being pursued in a number of trials currently but does present some methodological challenges (Goddard & Drummond, 1991). If the trial practice differs from routine practice then cost data collected during trials may not be relevant to subsequent clinical practice. Multi-centre trials will often involve a number of countries where cost implications may differ and the need for economic evaluation may alter the patient numbers required for adequate statistical power in a trial.

Full evaluation of cancer care is a very important area of research and requires careful application and further development of appropriate methods both in clinical trials and in clinical practice. Extensive collaboration between clinicians and economists will be necessary if Health Services, and ultimately patients, are to benefit from these approaches.

**References**

CALMAN, K.S., MCVIE, J.G. & SOUKOP, M. (1978). Cost of outpatient chemotherapy. *Br. Med. J.*, **1**, 493-494.

DE KONING, H.G., VAN INEVELD, B.M., DE HAES, J.C.J.M., VAN OORTMARSSEN, G.J., KLIJN, J.G.M. & VAN DER MASS, P.J. (1992). Advanced breast cancer and its prevention by screening. *Br. J. Cancer*, **65**, 950-955.

DRUMMOND, M.F. (1980). *Principles of Economic Appraisal in Health Care*. New York: Oxford University Press.

DRUMMOND, M.F., TEELING SMITH, G. & WELLS, N. (1988). *Economic Evaluation in the Development of Medicines*. London: Office of Health Economics.

GODDARD, M. & DRUMMOND, M.F. (1991). The economic evaluation of cancer treatments and programmes. *Europ. J. Cancer*, **27**, 1191-1196.

HURLEY, S.F., HUGGINS, R.M. & SYNDER, R.D. & BISHOP, J.F. (1992). The cost of breast cancer recurrences. *Br. J. Cancer*, **65**, 449–455.

MAGUIRE, P. & SELBY, P. (1989). Assessing quality of life in cancer patients. *Br. J. Cancer*, **60**, 437–440.

McILLMURRAY, M.B. & HOLDCROFT, P.E. (1993). Supportive care and the use of relaxation therapy in a district cancer service. *Br. J. Cancer*, **67**, 861–864.

MOSKOWITZ, M. (1987). Cost benefits determination in screening mammography. *Cancer*, **60**, 1680–1683.

PECKHAM, M. (1991). Research and development for the National Health Service. *Lancet*, **338**, 367–371.

RICHARDS, M.A., BRAYSHER, S., GREGORY, W.M. & RUBENS, R.D. (1993). Advanced breast cancer: use of resources and cost implications. *Br. J. Cancer*, **67**, 856–860.

SELBY, P. (1991). The role of the clinical editor. *Br. J. Cancer*, **63**, 1–2.

TWENTYMAN, P.R. & SELBY, P. (1991*a*). The process of peer review. *Br. J. Cancer*, **63**, 168–170.

TWENTYMAN, P.R. & SELBY, P. (1991*b*). The way ahead. *Br. J. Cancer*, **63**, 327–328.

TUCK, J., WALKER, A., WHYNES, D.K. *et al.* (1989). Screening and the costs of colorectal cancer. *Public Health*, **103**, 413–419.